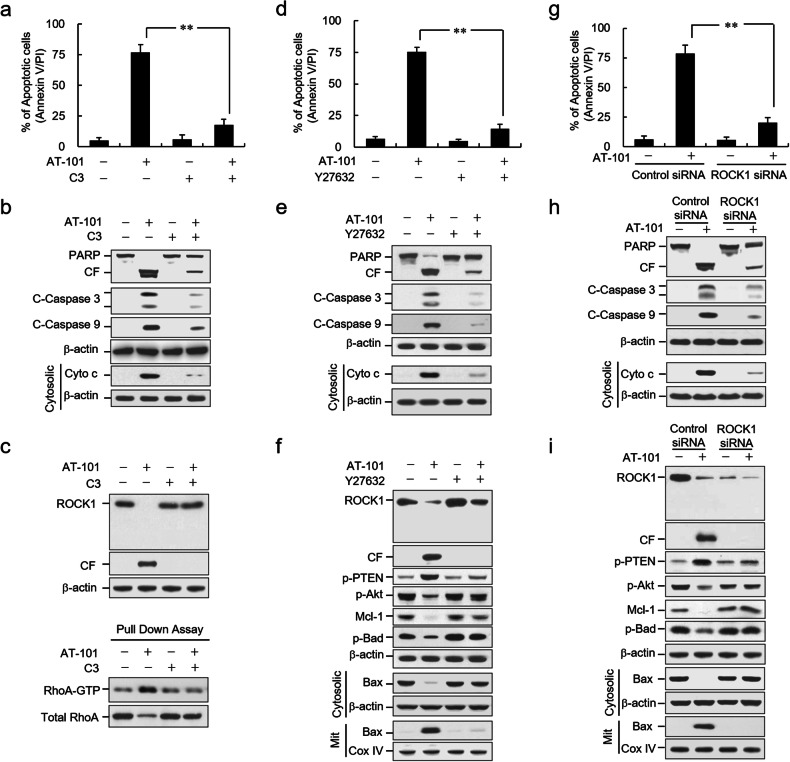# Correction to: RhoA/ROCK/PTEN signaling is involved in AT-101-mediated apoptosis in human leukemia cells in vitro and in vivo

**DOI:** 10.1038/s41419-025-07487-2

**Published:** 2025-04-03

**Authors:** G. Li, L. Liu, C. Shan, Q. Cheng, A. Budhraja, T. Zhou, H. Cui, N. Gao

**Affiliations:** 1 Department of Pharmacognosy, College of Pharmacy, Third Military Medical University, Chongqing, China; 2 Graduate Center for Toxicology, College of Medicine, University of Kentucky, Lexington, KY USA; 3 State Key Laboratory of Silkworm Genome Biology, Southwest University, Chongqing, China

Correction to: *Cell Death and Disease* 10.1038/cddis.2013.519, published online 16 January 2014

The authors reported errors in Figs. 4A, B and 6B. The correct figures are shown in the erratum file. The authors state that this correction does not affect the other results of Figs. 4 and 6, nor does it affect the conclusion of this article.

Incorrect Fig. 4
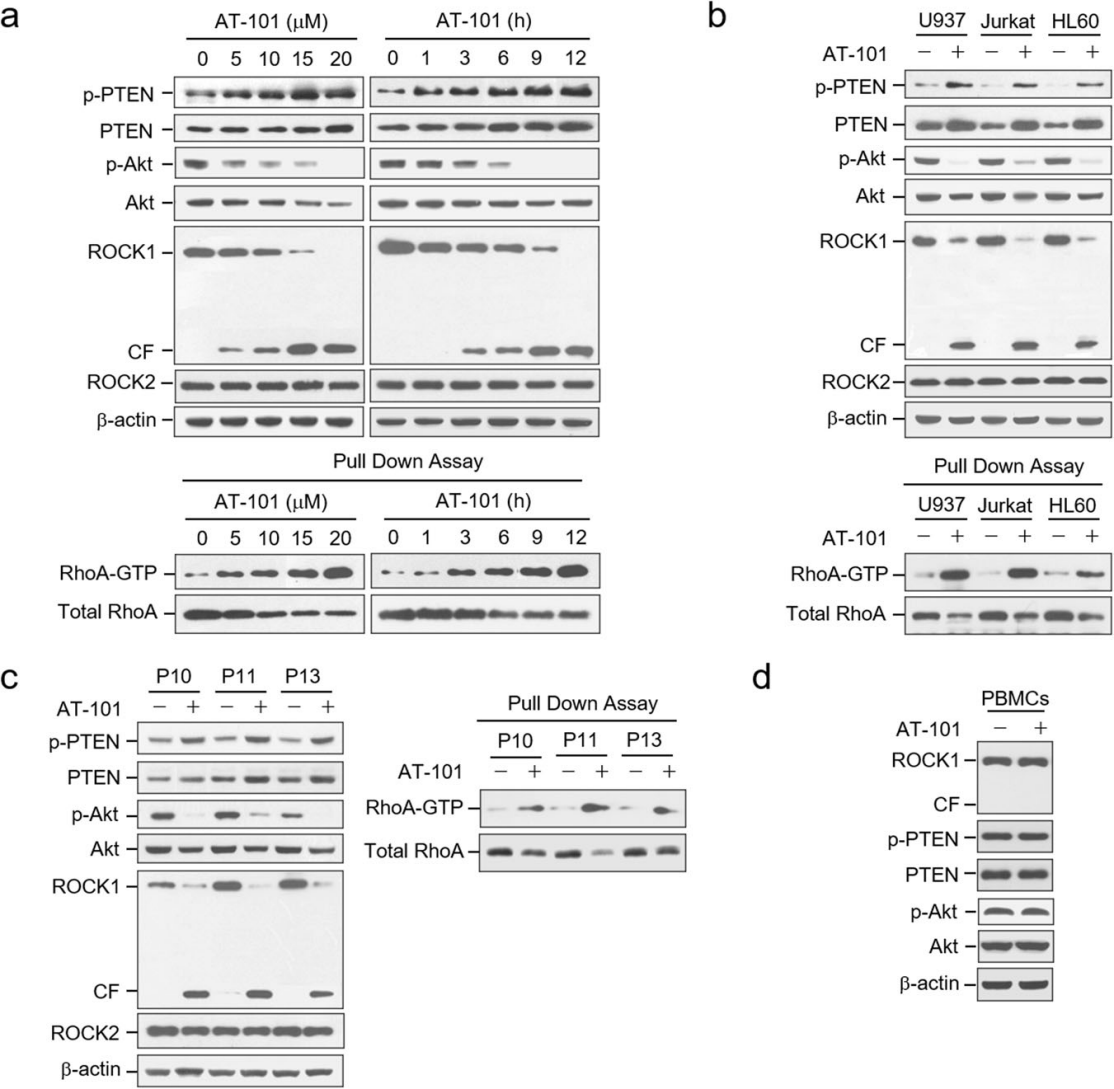


Corrected Fig. 4
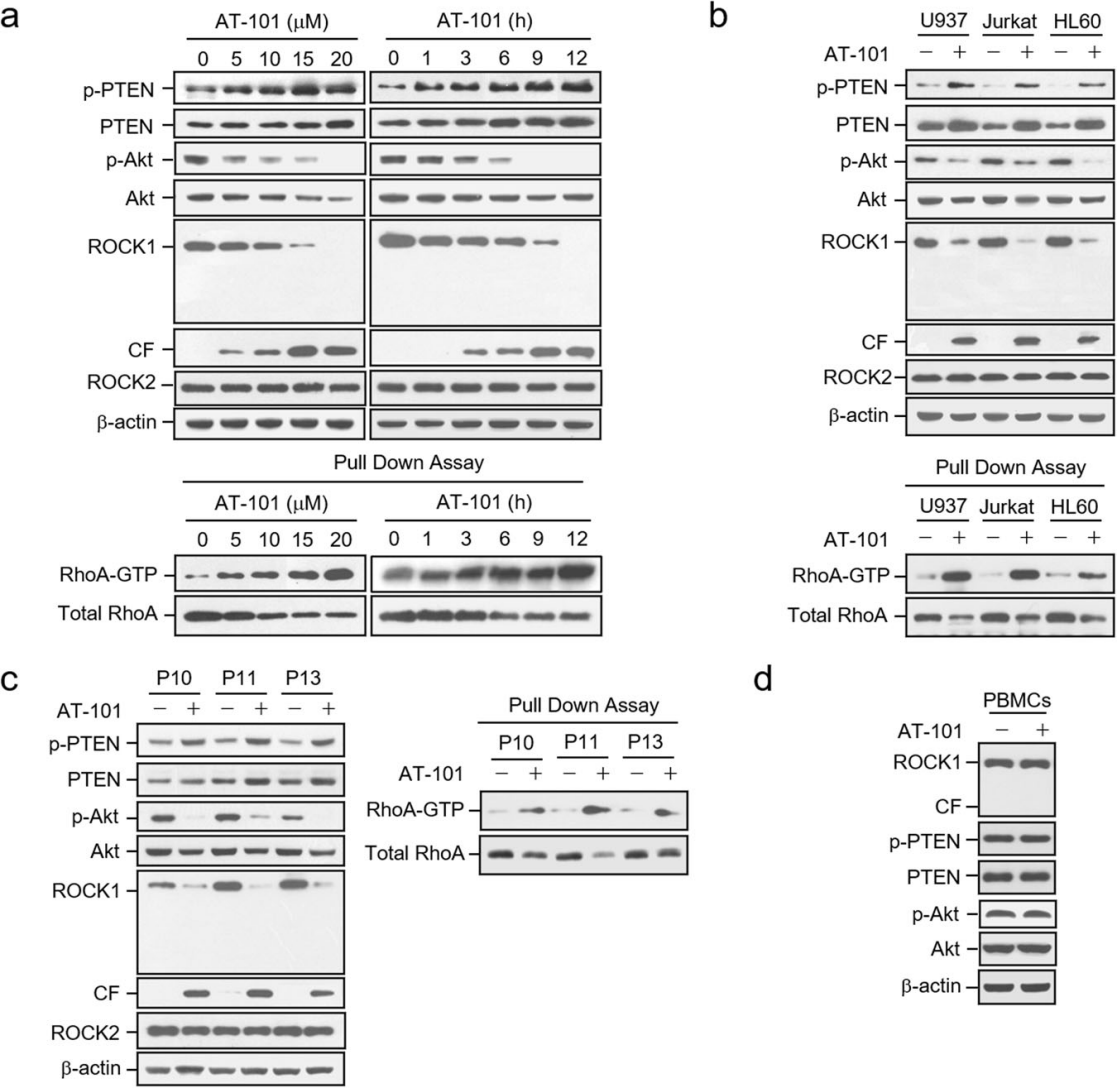


Incorrect Fig. 6
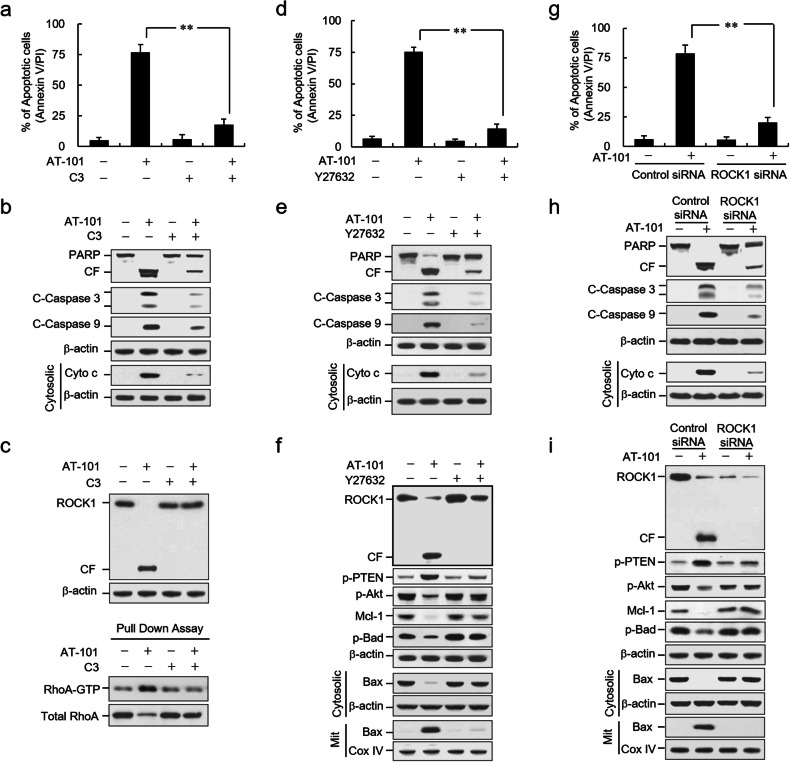


Corrected Fig. 6